# Cross-sectional, hospital-based analysis of headache types using ICHD-3 criteria in the Middle East, Asia, and Africa: the Head-MENAA study

**DOI:** 10.1186/s10194-023-01555-8

**Published:** 2023-03-13

**Authors:** H. Genc, B. Baykan, H. Bolay, D. Uluduz, I. Unal-Cevik, N. Kissani, O. Luvsannorov, M. Togha, A. A. Ozdemir, A. Ozge, on behalf of Head-MENAA study group, M. Cakan, AK Ak, F Celik, MO Orun, D Seker, A Kucuk, S Ozkan, M Kiraz, TC Sirin, R Ocal, HA Hakyemez, MO Yener, VA Serim, N Cinar, ED Unal, FM Domac, MF Ates, BG Turkoglu, G Gursoy, S Cekic, SK Aslan, D Agircan, AC Oktar, EA Demirel, P Gelener, EAA Ibrahim, A Evlice, G Gorken, ZS Sanli, BRH Bayır, N Tepe, T Okluoglu, TG Demir, MY Badr, D Vuralli, E Jafari, B Polat, A Ermis, E Khanmammadov, O Yolcu, B Kul, F Sakadi, S Ulutas, T Akturk, MT Ketema, S Lala, APSA Cedric, SK Velioglu, O Kirbasoglu, RR Moustafa, AG Nowar, SC Kabay, VK Gumanovna, YM Yifru, S Nasergivehchi, I Azizova, O Kizek, E Ekizoglu, EK Orhan, D Melka, B Alemayehu

**Affiliations:** 1 Van Training and Research Hospital, University of Health Sciences, Van, Turkey; 2 Istanbul Faculty of Medicine, EMAR Medical Center, Istanbul University, Istanbul, Turkey; 3 Faculty of Medicine, Department of Neurology and Algology, Gazi University, NOROM, Ankara, Turkey; 4 Medical Faculty, Department of Neurology, Istanbul University-Cerrahpaşa, Istanbul, Turkey; 5 Faculty of Medicine, Department of Neurology, Hacettepe University, Ankara, Turkey; 6 Neuroscience Research Laboratory in Marrakesh Medical School, Cadi Ayyad University, Marrakech, Morocco; 7 Department of Neurology, Mongolian National University of Medical Sciences, Ulaanbaatar, Mongolia; 8 Department of Neurology, Tehran University of Medical Sciences, Tehran, Iran; 9 Department of Biostatistics and Medical Informatics, University of Mersin, Mersin, Turkey; 10 Faculty of Medicine, Department of Neurology, Mersin University, Mersin, Turkey

**Keywords:** ICHD-3, Headache frequency, Neurology clinics, Migraine, MOH, Pain perception, Geographic regions, The Head-MENAA study

## Abstract

**Background:**

Headaches are frequent neurological disorders that are yet to be unveiled and treated comprehensively worldwide. Bearing in mind that the distribution of headache subtypes in neurology clinics (NC) is essential for planning appropriate diagnostic and therapeutic approaches, the primary goals of this multi-centric study are to carry out inter-regional comparisons by using current diagnostic criteria with evaluations of neurologists to delineate headache burden.

**Methods:**

A cross-sectional study between April 1 and May 16, 2022 was conducted with the participation of 13 countries from the Middle East, Asia, and Africa. Patients were included in the study on a specific day each week during five consecutive weeks. All volunteers over the age of 18 and whose primary cause for admission was headache were examined. The patients admitted to NC or referred from emergency services/other services were evaluated by neurologists by means of the International Classification of Headache Disorders (ICHD-3) criteria.

**Results:**

Among the 13,794 patients encountered in NC, headache was the primary complaint in 30.04%. The headache patients’ mean age was 42.85 ± 14.89 (18–95 years), and 74.3% were female. According to the ICHD-3 criteria, 86.7% of the main group had primary headache disorders, 33.5% had secondary headaches, 4% had painful cranial neuropathies along with other facial and headaches, and 5.2% had headaches included in the appendix part showing some overlapping conditions. While the most common primary headache was migraine without aura (36.8%), the most common secondary headache was medication-overuse headache (MOH) (9.8%). Headaches attributed to COVID-19, its secondary complications, or vaccines continue to occur at rates of 1.2%-3.5% in current neurology practice. Pain severity was significantly lower in Ivory Coast and Sudan than in Türkiye, Turkish Republic of Northern Cyprus, Iran, Egypt, Senegal, Tatarstan, and Azerbaijan (*p* < 0.001).

**Conclusions:**

The study showed that migraine is still the most common motive for admissions to NC in different regions. Furthermore, MOH, an avoidable disorder, is the most common secondary headache type and appears to be a significant problem in all regions. Remarkably, pain perception differs between regions, and pain intensity is lower in Africa than in other regions.

**Supplementary Information:**

The online version contains supplementary material available at 10.1186/s10194-023-01555-8.

## Introduction

Despite some regional differences, headache disorders are a global problem affecting people of all ages and races with different incomes and geographic regions [1]. In the 2019 Global Burden of Disease Study, migraine ranked second alone among the causes of disability and first in women under 50 years of age [2]. The estimated global prevalence of active headache disorder is 52% [3], and headache remains to be underestimated, under-recognized, and undertreated worldwide [1].

Information on the epidemiology of neurological disorders, especially in developing countries, is limited due to the limited resources and the need for trained health workers and neurologists [4]. Headache is the most common reason for referral to neurologists [5]. In order to optimize diagnostic and therapeutic approaches, it is crucial to know the distribution of patients with headaches among those seeking medical help in neurology clinics (NC) [6]. In addition to planning human resources such as physicians, effective training programs on headache should be prepared and regulation of health expenditures is to be allocated. In studies conducted at different times and in other geographic locations, differences in the prevalence of headaches are noteworthy. This may be due to many parameters such as methodological differences. Another variable is concerned with the researchers being neurologists or not; and the use of different International Classification of Headache Disorders (ICHD) criteria in previous studies [3]. With this respect, evaluation of the diagnosis of a neurological disorder by healthcare professionals other than a qualified neurologist or questionnaire-based studies may lead to diagnostic mistakes [4].

The aim of this study is to determine the distribution of headache in different service areas in NC according to the ICHD-3 criteria [7] through neurologists. The secondary aim is to update headache distributions with the ICHD-3 criteria and to identify all headache subtypes in admitted patients with headache since the previous studies were generally performed in accordance with the old ICHD-I or ICHD-II criteria. Finally, the variation of headache subtypes is scrutinized according to the locations where the research was conducted and the possible factors that lead to the difference in findings.

## Methods

This study was designed as a multinational, multicentered and cross-sectional. The patients were admitted to the study on a particular day each week for five consecutive weeks between April 01 and May 16, 2022. The days of the study were selected using the “Research Randomizer Program”. Trained neurologists evaluated all patients. Prior to the study, all researchers underwent a reconstructed briefing about the ICHD-3 criteria. Current diagnostic criteria were applied for headaches attributed to Coronavirus disease 2019 (COVID-19), headaches attributed to complications secondary to COVID-19, and headaches attributed to the COVID-19 vaccine. All volunteer patients over 18 years old and whose primary reason for admission was headaches were included in the study. The researchers received online informed consent forms from all volunteered patients. Patients not reporting headaches at admission, being younger than 18 years, and did not agree to participate in the study were excluded. A structured, standardized questionnaire was applied to the volunteers participating in the study (Head-MENAA Study Questionnaire-Supplement 1). The neurologists coded headache subtypes in the same questionnaire according to the ICHD-3 criteria. We added options for headaches attributed to COVID-19, complications secondary to COVID-19, and the COVID-19 vaccine. The last part was left open-ended for diagnoses in the appendix that could be included in the main text of the classification later.

**Fig. 1:**
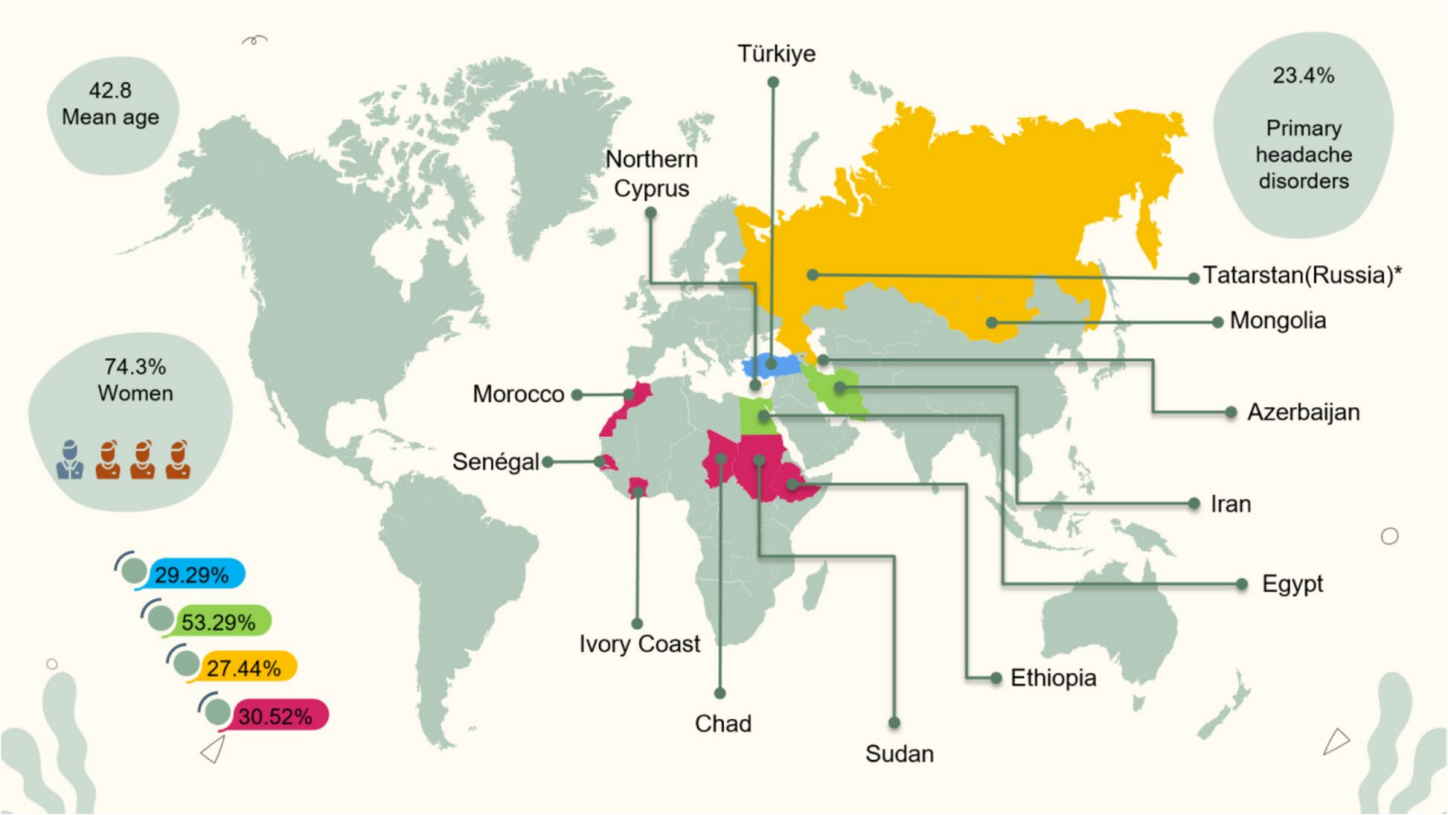
Prevalences of headaches in the participating Neurology Clinics in the Middle East, Asia, and Africa (at the bottom left corner)

The invitations were sent to 32 countries from the Middle East, Asia, and Africa for their involvement in the study. Due to various intervening factors (such as Ramadan, summer vacation, and failure to obtain ethics committee approval within the specified time), 70 researchers from 13 countries were able to take an active part in the research. Since 83% of the patients participating in the study were from Türkiye, Türkiye was evaluated separately to analyze the data more accurately. Ivory Coast, Chad, Senegal, Sudan, Ethiopia, and Morocco from Africa; Egypt and Iran from the Middle East; Tatarstan, Turkish Republic of Northern Cyprus, Azerbaijan, and Mongolia from Asia joined the study (Fig. 1). The acronym of the study was determined as Head-MENAA (Middle East, North Africa, Asia) by using the initials of the names of the regions participating in the study. Ethics committee approval was obtained by the study coordinator (H.G.) from the University of Health Sciences, Van Training and Research HospitalClinical Research Ethics Committee (Decision no: 2022/05–01, Date: March 02, 2022). The researchers registered all volunteer patients whose main motive was headache in their visits. The study included the patients arriving on the same day from outpatient clinics, neurology service, private clinics, and emergency/other service consultations. Headaches were categorized under four main headings as primary headaches (Part I), secondary headaches (Part II), neuropathies & facial pains (Part III), and appendix (Part IV) (Table 1). Headaches attributed to COVID-19, headaches attributed to complications secondary to COVID-19, and headaches attributed to the COVID-19 vaccine were evaluated in the appendix. This article contains the initial analysis results of the Head-MENAA study.

**Table 1:** Distribution and clinical characteristics of headache according to Part I-II-III and Appendix categories in ICHD-3

	Part I	Part II	Part III	Appendix
**The total number of headache patients per ICHD-3 groups (%)**^a^	3226 (86.7%)	1246 (33.5%)	149 (4%)	199 (5.3%)
**Türkiye (%)**	2676 (71.9%)	1036 (27.8%)	118 (3.1%)	156 (4.1%)
**The Middle East (%)**	199 (5.3%)	77 (2%)	93 (2.6%)	14 (0.4%)
**Asia (%**	116 (4.5%)	54 (1.5%)	8 (0.2%)	11 (0.3%)
**Africa (%)**	235 (6.3%)	79(2.1%)	14 (0.3%)	18 (0.5%)
**Gender (Female %)**	75.2%	73%	72.5%	73.9%
**Age (Years: Mean + SD)**	42.78 ± 14.82	43.3 ± 14.95	42.99 ± 16.16	42.63 ± 15.59

^a^There were more than one diagnoses in 36.1% of patients

### Statistical analysis

Normality control of continuous variables was done with the Shapiro–Wilk test. Parametric tests were used for the variables that fit the normal distribution, and non-parametric tests were used for the ones that did not. Independent Sample t-test and Mann Whitney U test were applied for the comparison of two independent groups. One-Way ANOVA and Kruskal Wallis tests were used to carry out the comparative analysis of more than two groups. In addition, Tukey was used as the post-hoc test. The chi-square test was applied in the analysis of categorical data. The analysis of the data was carried out through the Statistica 13.5.0.17 program. The statistical significance level was taken as p ≤ 0.05.

## Results

Overall, headache was the primary referral cause of 30.04% of the 13,794 patients evaluated by the neurologists in the study. 81% of the patients were assessed in neurology outpatient clinics, 10% in private offices, 2% in emergency services, 3% through consultation in other services, 2% in general outpatient clinics, and 2% in other clinics. The number of patients taking part in the study was 3091 from Türkiye, 26 from Ivory Coast, 33 from Chad, 27 from Senegal, 54 from Sudan, 60 from Ethiopia, 62 from Morocco, 74 from Egypt, 151 from Iran, 17 from Tatarstan, 56 from the Turkish Republic of Northern Cyprus, 16 from Azerbaijan, and 55 from Mongolia. A total of 422 patients (377 from Türkiye, 2 from the Middle East, 5 from Asia, and 38 from Africa) refused to participate in the study.

Headache rates observed in NC in the Middle East, Asia, and Africa have been shown in Fig. 1.

The mean age of the participants was 42.85 ± 14.89 (18–95 years), and 74.3% were female patients. The number of painful days in the previous month was 11.73 ± 10.41, and the lifetime painful period was 5.69 ± 8.34 years. Pain intensity measured by numbered visual scale (NVS) was 7.03 ± 1.73. Combined headache types (more than one subtype of headache diagnosis) were present in 36.1% of the patients. Primary headache disorders (Part I in ICHD-3) comprised 23.4% of all evaluated patients in NC, and 86.7% of all the patients admitted with headaches. Among patients with a primary headache disorder, 75.2% were female. The distribution of headache rates by diagnostic categories, regions, and demographic characteristics is given in Table 1. According to the ICHD-3 criteria of patients with headaches, 33.5% had secondary headaches (Part II), 4% of the subjects had painful cranial neuropathies, facial pain and other headaches (Part III), and 5.3% had other headache subtypes in the Appendix as well as the headaches attributed to COVID-19, headaches attributed to complications secondary to COVID-19, and headaches attributed to the COVID-19 vaccine (displayed in the Appendix of Table 1. In male patients with headaches, secondary headaches were more common than primary headaches (*p* = 0.004). There was no difference between primary and secondary headaches regarding age, gender, and pain intensity. However, lifetime pain duration in primary headaches was 5.80 ± 8.40 years, and it was longer than secondary headaches (4.51 ± 7.20 years) (*p* = 0.036).

**Fig. 2:**
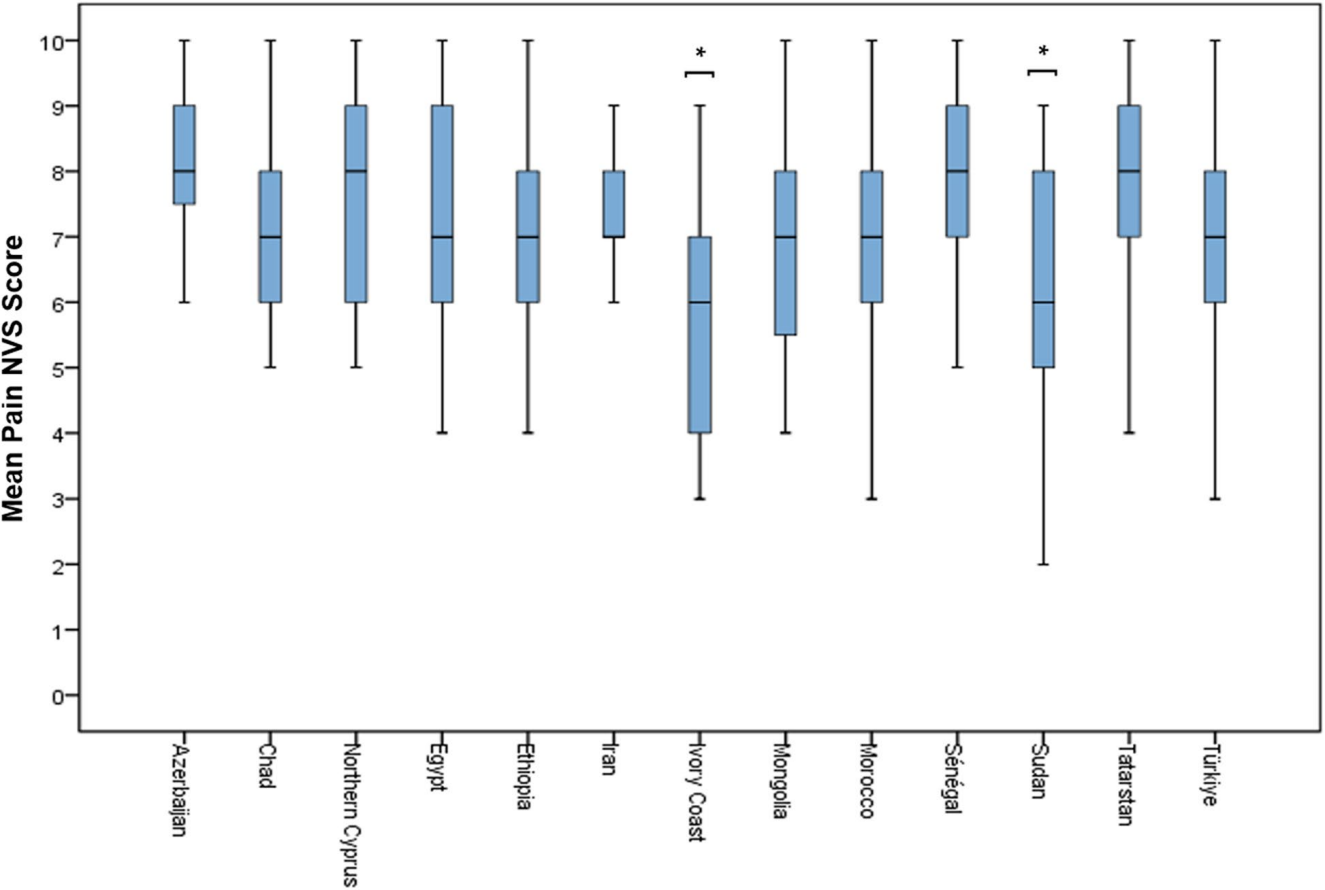
Distribution of mean severity of headache according to countries (*different than Türkiye, Turkish Republic of Northern Cyprus, Iran, Egypt, Senegal, Tatarstan, and Azerbaijan, *p* < 0.001)

Headache severity measured with NVS seems to differ according to the regions. Pain intensity was significantly lower in the African region compared to the other regions (*p* < 0.001). When analyzed separately according to the countries, pain severity was significantly lower in Ivory Coast and Sudan than in Türkiye, Turkish Republic of Northern Cyprus, Iran, Egypt, Senegal, Tatarstan, and Azerbaijan (Fig. 2). There was a significant difference among the regions regarding lifetime pain duration (*p* < 0.001). Total lifetime pain duration was 5.84 ± 8.43 years in Türkiye while it is 7.57 ± 10.30 years in the Middle East; 4.71 ± 7.62 years in Asia; and 2.80 ± 3.93 years in Africa. Unlike the lifetime pain duration being significantly longer in the Middle East than in the other regions, it is seen that patients in Turkey are likely to suffer from pain longer in Türkiye than in Africa (*p* < 0.001). While the mean lifetime pain duration of both primary and secondary headaches was substantially more prolonged in the Middle East than in the other regions, it was longer in Türkiye than in Africa (*p* < 0.001). The severity of primary headaches was milder in Africa when compared to the other regions (*p* < 0.001). The severity of secondary headaches in Africa was again found to be significantly lower than the regions of Türkiye and Asia (*p* < 0.013).

**Fig. 3:**
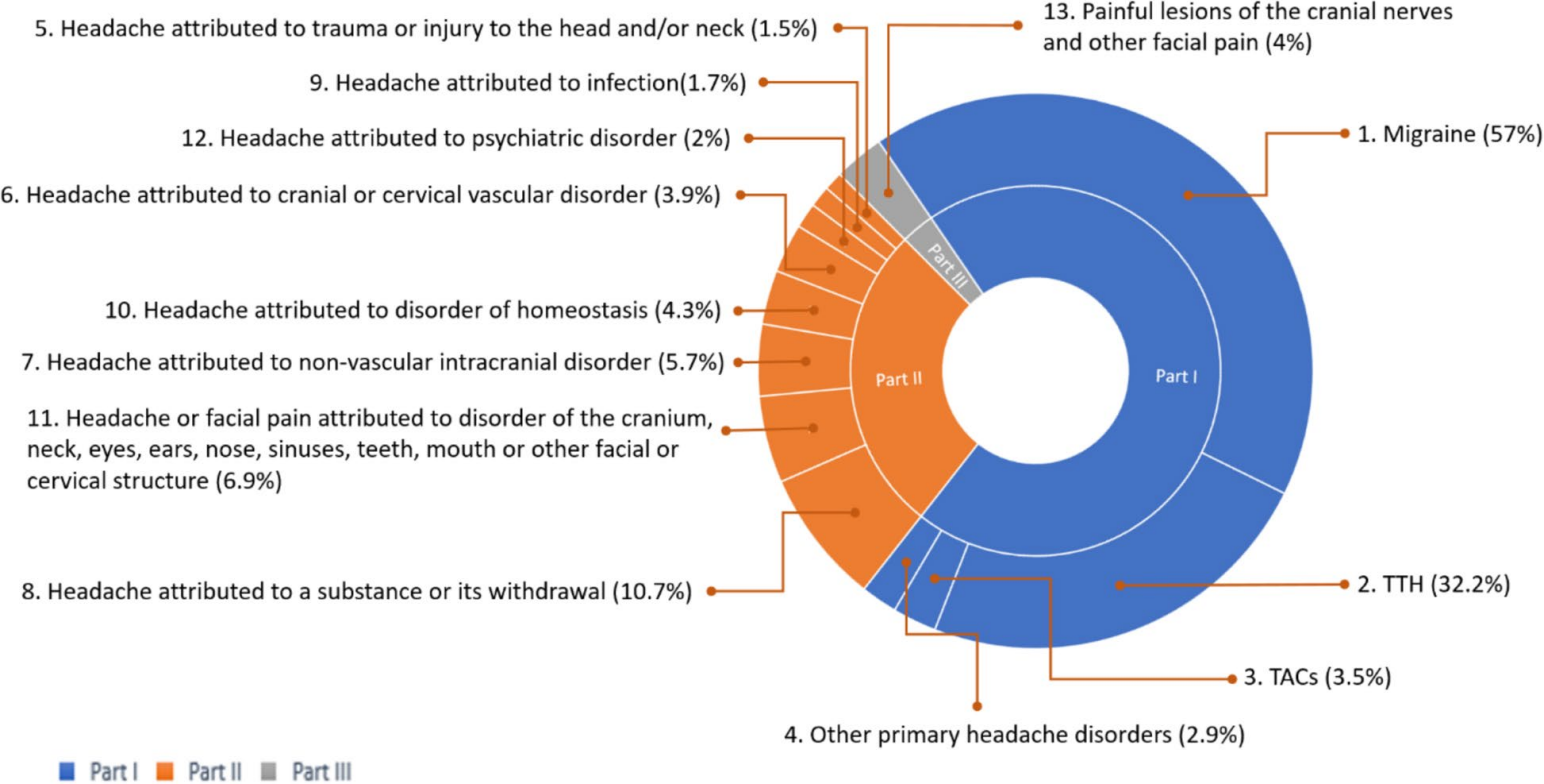
The general distribution of headache types

**Fig. 4:**
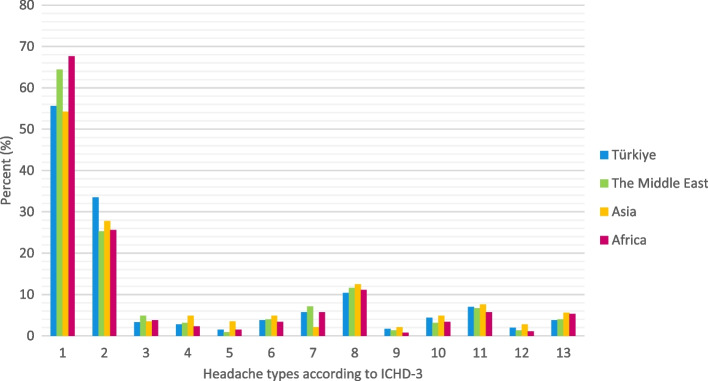
Distribution of headache types according to regions (1; Migraine, 2; TTH, 3; TACs, 4; Other primary headache disorders, 5; Headache attributed to trauma or injury to the head and/or neck, 6; Headache attributed to cranial or cervical vascular disorder, 7; Headache attributed to non-vascular intracranial disorder, 8; Headache attributed to a substance or its withdrawal, 9; Headache attributed to infection, 10; Headache attributed to disorder of homeostasis, 11; Headache or facial pain attributed to disorder of the cranium, neck, eyes, ears, nose, sinuses, teeth, mouth or other facial or cervical structure, 12; Headache attributed to psychiatric disorder, 13; Painful lesions of the cranial nerves and other facial pain)

The general and regional distribution of headache types among patients with headaches according to the ICHD-3 criteria and categories is summarized in Figs. 3 and 4, respectively.

The most common headache subtype among patients with headaches evaluated in NC was migraine without aura (36.8%). The most common secondary headache diagnosis was medication-overuse headache (MOH) (9.8%). The most common combination of headaches was between migraine and MOH with a rate of 5.7%. MOH headache was observed in 10% of patients with migraine and 11.9% of patients with TTH. Migraine and TTH were observed in an integrated manner in 5.53% of patients with headaches. The mean ages in migraine and TTH patients were 42.33 ± 14.53 and 43.79 ± 15.20. The mean age of onset of headache was 36.08 ± 15.70 in migraine patients and 38.33 ± 16.35 in TTH patients. The distribution of migraine and TTH according to the regions was specified in terms of frequency, age, gender, headache frequency, severity, and lifetime pain duration in Table 2.

**Table 2:** Distribution of migraine and TTH according to regions in terms of frequency, age, gender, headache frequency and severity, and the lifetime pain duration

	Türkiye	The Middle East	Asia	Africa	p
**Migraine (%)**	56.3%^ab^	64.7%^b^	54.5%^a^	69.1%^a^	** < 0.001**
The mean age (years)	42.45 ± 14.82	41.09 ± 13.34	43.55 ± 13.57	41.60 ± 13.70	0.549
Female ratio (%)	76.50%	72.40%	71.80%	68.90%	0.098
Frequency of headache	11.77 ± 9.67^ab^	14 ± 10.36^a^	9.14 ± 8.23^a^	6.18 ± 8.15^ab^	** < 0.001**
Severity of headache	7.21 ± 1.68^a^	7.28 ± 1.55^a^	7.42 ± 1.59^a^	6.61 ± 1.62^a^	** < 0.001**
Lifetime pain duration	6.38 ± 8.71^a^	8.10 ± 10.51^a^	5.32 ± 9.59	2.82 ± 3.92^a^	** < 0.001**
**TTH (%)**	33.5%^a^	25.3%^a^	27.8%	25.6%^a^	**0.003**
The mean age (years)	44.13 ± 15.23	39.51 ± 12.56	43.82 ± 15.05	41.87 ± 16.46	0.119
Female ratio (%)	76.1%^a^	68.4%^a^	57.5%^a^	71.6%^a^	**0.03**
Frequency of headache	12.29 ± 10.15^a^	13.74 ± 10.52^a^	11.70 ± 9.08^a^	5.76 ± 8.68^a^	** < 0.001**
Severity of headache	6.83 ± 1.76	7.40 ± 1.64^a^	6.82 ± 1.75	6.41 ± 2.13^a^	**0.024**
Lifetime pain duration	5.67 ± 8.33	6.69 ± 10.10	3.86 ± 3.79	3.59 ± 4.48	0.098

Each subscript letter denotes a subset of categories whose column proportions/means do differ significantly from each other at the 0.05 level

The migraine related headaches are more common in Africa than in Türkiye and Asia and also are encountered more in the Middle East than in Türkiye (*p* < 0.05). The findings in the analysis are found to be significant. The mean age of onset of headache was 36.08 ± 15.70 in migraine patients, and in terms of gender there was no difference across the regions. It was statistically significant that the migraine headache frequency was higher in the Middle East than in all regions, and the frequency in Türkiye was more than in Africa (*p* < 0.001). The headache severity was lower in Africa than in all regions, which was statistically significant (*p* < 0.001). Lifetime pain duration was significantly shorter in Africa than in the Middle East and Türkiye (*p* < 0.001) (Table 2).

The TTH ratio, higher in Türkiye than in Africa and the Middle East, was statistically significant (*p* = 0.003). Besides, it was statistically significant that TTH was more common in women in Türkiye, the Middle East, and Africa than in Asia (*p* = 0.03). The mean age of onset of headache was 38.33 ± 16.35 in TTH patients. It was statistically significant that the TTH headache frequency was less in Africa than in all regions (*p* < 0.001). Headache severity was lower in Africa than in the Middle East (*p* < 0.024), and there was no difference across the regions regarding lifetime pain duration (p  =0.098) (Table 2).

In the last part, 5.2% of the patients with headaches were diagnosed with the headaches attributed to COVID-19, headaches attributed to complications secondary to COVID-19, and headaches attributed to the COVID-19 vaccine as stated in the criteria of the appendix part of ICHD-3. Among all headache patients, headaches attributed to COVID-19 were observed in 3.5% meanwhile the headaches attributed to complications secondary to COVID-19 were observed in 1.2%, and finally the headaches attributed to the COVID-19 vaccine were observed in 1.5%. The diagnoses included in the appendix were not thoroughly specified; general criteria were sufficient to make a diagnosis among all physicians. There was little need for additional criteria (0.32%), and the criteria specified were A11.2.4 Headache attributed to upper cervical radiculopathy, A12.4 Headache attributed to separation anxiety disorder, A12.5 Headache attributed to panic disorder, A12.9 Headache attributed to a post-traumatic stress disorder, A5.8 Acute headache attributed to other trauma or injury to the head and/or neck. According to the ICHD-3 criteria, no significant difference was observed in all the regions regarding the incidence of headache subtypes except migraine and TTH.

## Discussion

Headache disorders remain to be under-recognized and undertreated especially in developing populations [1, 8–12]. The patients examined by neurology specialists from 13 countries took part in the study. Overall, 30.04% of the patients were admitted due to headache complaints, and there were regional differences. In Türkiye, the 29.29% rate was lower compared to 42.8% [13] and 35.1% in outpatients [14], reported in previous studies conducted in Türkiye in 2012 and 2007, respectively. The headache rate among all patients in the Middle East was 53.29%, which was significantly higher than in other regions and could not be compared with previous studies due to insufficient data. In our study, this rate was 27.44% in the Asian region similar to the findings of a study conducted in Bangladesh (24.7%) [15]. On the other hand, the frequency obtained with limited country data from Asia is higher than previously reported figures from China (19.5%) [11], Saudi Arabia (15.5%) [16], and Thailand (9.8%) [17]. In this study the similar results were obtained in Africa with a rate of 30.52%. In studies conducted in some countries in the African continent, the rates ranged from 1.6–31.9% [4, 18–20]. For example, it was reported as 1.6% in Ghana, which was low because primary headaches were usually managed by primary care physicians [18]. Headache rates in Zimbabwe and Zambia were 11.4% [4] and 19.4% [19], respectively, and lower rates might be due to a lack of neurologists and diagnostic aid resources [4]. Similar to our study, the prevalence of headaches in NC in Cameroon was 31.9% [20]. The factors that increase the reliability of the frequencies in our study are the evaluation of patients by neurologists, preliminary training on diagnostic criteria, active communication throughout the project, and further analysis that is not limited to outpatients.

In our study, the female-male (F: M) proportion was 2.9:1 in patients who applied to the outpatient clinic with headaches, and there was no difference with respect to the regions regarding gender distribution (Fig. 1). This rate is close to the findings of the previous studies [14, 13]. In the study executed in Zimbabwe, the F: M proportion was 1.8:1 lower than this study, yet the female dominance was striking [4]. This female dominance in neurology clinics does not reflect the gender distribution in the general population [14, 21]. The reason is that the willingness of men to seek help for headache treatment was twice less than that of women [22] as the research revealed. The mean age of the patients in our study was similar to the studies performed in Asia and Africa [4, 13].

In our study, the lifetime painful duration was 5.69 ± 8.34 years. In a study conducted in Burkina Faso, this was 4.37 ± 2.72 years, and 61.8% of patients suffered from headaches that lasted longer than six months [23]. Our patients with headaches reported that they experienced headaches in one-third of a month reflecting the burden on their social and personal lives.

The mean NVS score reflecting the severity of headache in our patients was high (7.03 ± 1.73). The pain was highly severe in 18% of the patients, severe in 47%, moderate in 34%, and mild in 1%. Severe pain rates reported in previous studies were 9.3% in China [11], 41.5% in Türkiye [14], and 59.6% in India [10]. In Burkina Faso, the pain was very severe in 14.7% of patients with headaches, severe in 41.2%, moderate in 31.4%, and mild in 12.7% [23]. These differences may be due to age, gender, sample size, the difference in pain perception, pain scales used, and genetic differences. For example, in the study conducted in Burkina Faso, the mean NVS score was 4.8 ± 2.9 in male patients and 6.6 ± 3.7 in female patients. While the mean NVS score was 6 ± 3.2 in patients aged 5–39, it was 5 ± 3 in patients 60 years and older [23]. In the study of Ho and Ong, non-Chinese people in Singapore suffered from more severe headaches than the Chinese population [24].

Pain perception and response mechanisms differ among individuals [25] and are related to many different factors along with culturally differentiated coping mechanisms [26]. In interracial pain perception studies, African Americans (AA) generally reported greater pain severity/intensity than the other races, but the clinical manifestation may differ [27]. For example, in a study conducted on AAs with chest pain, the patients were more likely to underestimate their pain and were also less likely to report it [28]. This condition often delays emergency care [29]. Therefore, clinicians may tend to underestimate and misinterpret the presence and intensity of pain in AAs [30]. In our study, pain intensity was lower in the African region compared to the other areas. This result may reflect the possibility that patients refer to a health clinic earlier due to their low tolerance to pain. On the contrary, although patients have more severe pain, it can also be perceived as a feeling of inferiority. It may be due to the inability to express pain adequately as a result of language problems or because clinicians underestimate the severity of pain. These results obtained from few centers in Africa need to be investigated.

In our study lifetime pain duration of the patients admitted to NCs was significantly longer in the Middle East than in the other regions. The duration of pain is longer in Türkiye than in Africa. The differences in lifetime pain duration can be caused by various factors such as sociocultural, economic, and educational differences. Mechanisms of coping with pain and the duration may indirectly be reflected in personal and cultural differences. For example, a study performed on older Korean women revealed that patients associated a different meaning to pain. According to them, chronic pain is an inevitable consequence of aging and is not considered a problem to be solved [31]. South African researchers found that a large proportion of the population on the African continent uses traditional medicine as their primary source of health care [32]. Therefore, the widespread use of conventional remedies may interfere with headache symptoms and diminish the possibility of referrals to the NCs. Some studies have suggested that patients from historically disenfranchised cultures, such as Africa, are more resilient to pain and have lower expectations of pain and distrust of Western biomedical interventions for treatment. Similarly, such patients probably have socioeconomic disadvantages that hinder care access [33, 34]. In order to better analyze these results in our research, there is a need for additional information acquired from cross-racial studies including socioeconomic status, education, and cultural characteristics. In terms of healthcare providers’ education and service procedures, cultural perspectives of the patients, their belief and attitudes towards perception of pain and management should be taken into account [35].

### Primary headaches

Among patients with headaches, the rate of primary headaches was 50.1% in China [36], 52.9% in Burkina Faso [23], and 78.1% in Iran [8]. In our study, the rate of primary headaches among patients was significantly higher than in previous studies with 86.7% of patients with headaches in NC.

As far as the global prevalence estimate of TTH is 22%, that of migraine is 15% [37] is concerned, the disease burden, particularly in chronic disorders such as migraine can vary considerably by geography and ethnicity [38]. For example, in Mongolia, the crude 1-year prevalence of TTH was 29.2% and that of migraine was 24.1% [39]. On the other hand, the migraine burden in Africa is predicted to increase by more than 10% over the next decade [40]. Despite the prevalence and impact of migraine and TTH, they are still diagnosed in less than half of patients and are more neglected in those with comorbid diseases [11, 22, 41, 42]. Although it has been reported that TTH is more common than migraine in population-based studies, migraine is widely encountered reason for referral to specialists than TTH [43–45]. Migraine headache was observed more frequently than TTH with rates ranging from 41.6% to 80.8% in neurology clinics in prior studies [8, 46]. In our research, migraine headache was observed more frequently in Africa than in Türkiye and Asia, and in the Middle East than in Türkiye. Some studies showed a higher prevalence of migraine in Caucasians compared to African and Asian Americans [47, 48]. A meta-analysis also showed that the prevalence of migraine in Asia and Africa is lower than in Europe [49]. Although the social prevalence of TTH is higher than migraine, the higher prevalence of migraine in neurology outpatient clinics (NOC) is likely to stem from the severity of pain in migraine which is so high that it requires treatment [50]. In addition, TTH is often thought of as a “normal” headache, and many patients self-treat themselves with over-the-counter medications [51]. TTH has been observed more frequently in Türkiye than in Africa and the Middle East (Table 2). Contrary to the higher incidence of migraine in NOCs, TTH was observed more frequently than in Kuwait, with 29% versus 28.5% [52], and in Burkina Faso, with a rate of 27.5% versus 20.6% [23]. However, migraine was the most common primary headache (54.2–67.6%) in all regions in our study, and this was followed by TTH headache, with a rate of 25.3–33.5% (Table 2). In a study conducted in Africa, TTH was observed in 20.4% of patients [23] ranging from 24–34.1% in Asia [8, 11, 46, 53]. In both studies conducted in Türkiye, the TTH rate was found to be 28.9% [14] and 71.5% [13]; the high rate in the second study may be due to the study methodology. In our research, migraine and TTH were observed in combination in 5.53% of patients with headaches. The prevalence of migraine ranges from 11.3% to 14.4% in women and 3.6–6.7% in men in the studies conducted in Asia [54]. In a prevalence study conducted in Türkiye, the prevalence of migraine was 21.8% in women and 10.9% in men [55]. While the ratio of F:M was 2.8:1 in a study conducted on NOCs in Türkiye [14], it was 2.6:1 in India [53]. These results resemble the findings obtained in our study. In this study, the mean age in patients with migraine was 42, while the mean age of onset of headaches in patients with migraine was 36. Migraine had the highest prevalence in East Asia for adult women aged between 30 to 49 [9]. In the studies conducted in NOCs, the mean age was 39.2 in Türkiye [14] and 42.1 in China [36], which are similar to our research. TTH, like migraine, is also more common in women and middle-aged adults [23, 36, 46, 56]. In both studies carried out in NOCs of India, the mean age of migraine patients was younger, 27 and 33.6 years [53, 56]. In our research, the mean age of onset was 38, while the mean age was 43 for TTH patients. The tendency of TTH to be observed more in women is less than in migraine [57]. In our study, the F: M ratio in patients with TTH was 3.1:1 in Türkiye, 2.6:1 in the Middle East, and 2.5:1 in Africa, and it was approximately 1.4:1 with slight female dominance in Asia. In the study performed in NOCs in China, F: M was 2.2:1 in patients with TTH [37]; this outcome is highly close to the studies in Pakistan and India [46, 56]. The mean age in patients with TTH ranged from 40 to 47 in studies of patients in NOC in China and India which is similar to our study [11, 36, 56]. In contrast, another study from India found that the mean age for TTH was younger (25 years) [53]. The lifetime pain duration in migraine patients was approximately six years in Türkiye, eight years in the Middle East, five years in Asia, and three years in Africa (Table 2). In a study conducted in Türkiye in 2005, the mean lifetime headache duration in migraine patients in NOCs was 9.4 years [14]; in our research, this was meanly 6.16 years in all regions, 6.38 years for Türkiye. This striking result was attributed to the positive effects of awareness and education activities on physicians and patients. Furthermore, while the average number of painful days was 7.9 in the study conducted in Türkiye [14], this rate was generally 11.38 days in our research and 11.78 days in Türkiye. This may reflect that patients with mild and low-frequency headaches often try to cope with the pain independently. A study conducted in South Korea showed that only a quarter of individuals with migraines applied to a doctor for their headaches, 64.3% of them took medication for headaches, and most of them used over-the-counter medication [58]. A study in East Asia showed that many migraine patients were not diagnosed and did not consult a doctor for treatment. This suggests that migraine is still underdiagnosed and treated [9].

TACs were detected at a rate of 3.5%, and other primary headache disorders were measured at a rate of 2.9% by neurologists in our study. It was remarkable that there was no significant difference across the regions.

### Secondary headaches

Secondary headaches were found at a rate of 20.1% in Iran [8] and in Burkina Faso at 47.1% [23]. The higher incidence of secondary headaches in Africa is primarily due to tropical neuro infections [40]. When compared to Iran, the higher rate obtained in this study may be due to the fact that the physicians included the patients who had been examined in the emergency and other services besides outpatients in accordance with the methodology. The frequency of secondary headaches in our research and their comparison with previous studies are given in Table 3. In this study, the frequency of secondary headaches was not found to differ across the regions.

**Table 3:** Distribution of secondary headache types according to the results of the current study and the reflections of prior studies

	The results of current study					The results of some prior studies				
**The Secondary Headaches (ICHD-3 Criteria)**	**Total (%)**	**Türkiye (%)**	**The Middle East (%)**	**Asia (%)**	**Africa (%)**	**Türkiye(%)** [13]	**India (%)** [53]	**Burkina Faso (%)** [23]	**Iran (%)** [8]	**Ghana (%)** [18]
5.Headache attributed to trauma or injury to the head and/or neck	1.5	1.5	0.9	3.5	1.5	0.09	2.8	7.8	−	−
6.Headache attributed to cranial or cervical vascular disorder	3.9	3.8	4	4.9	3.4	3.01	−	−	2.8	13.7
7.Headache attributed to non-vascular intracranial disorder	5.7	5.7	7.1	2.1	5.7	1.24	4	5.9	−	−
8.Headache attributed to a substance or its withdrawal	10.7	10.4	11.6	12.5	11.1	4.22	1	−	−	−
9.Headache attributed to infection	1.7	1.7	1.3	2.1	0.8	0.1	0.7	2.9	1.8	−
10.Headache attributed to disorder of homeostasis	4.3	4.4	3.1	4.9	3.4	4.39	−	−	2	−
11.Headache or facial pain attributed to disorder of the cranium, neck, eyes, ears, nose, sinuses, teeth, mouth or other facial or cervical structure	6.9	7	6.7	7.6	5.7	4.43	4.1	14.7	1.8	−
12.Headache attributed to psychiatric disorder	2	2	1.3	2.8	1.1	11.4	−	−	4	−

The prevalence of Post-traumatic headaches is estimated to be approximately 4% of all symptomatic headaches [59]. Generally, the rate detected in NOCs is lower than this. For example, in a study conducted in Burkina Faso, post-traumatic headache was measured as significantly higher at 7.8% [23]. Albeit it was found to a lesser extent in the African region in our study. This rate in the previous studies may be due to sociocultural reasons, pain threshold, and accident rates. For example, the risk of death from road traffic accidents is the highest in the African Region (26.6 per 100,000 population) and the lowest in the European Region (9.3 per 100,000 population) [60].

Another critical issue is “Headache due to substance (use) or withdrawal.” The population prevalence of MOH, a sub-title of this group, is approximately 1% [61, 62] and becoming a growing problem worldwide [63]. In a study conducted in Mongolia, MOH’s age- and gender-adjusted prevalence was 5.7% [39]. MOH was the third most common cause of headaches in a survey among family physicians [64]. Similarly, in our study, MOH was the third most common cause of headaches and the most common secondary headache. As there is limited number of studies on MOH rates, more research is to be conducted on the prevalence of MOH among the races.

The epidemiology of headaches in Africa involves more secondary headaches primarily due to tropical neuro infections such as malaria and meningitis [40]. However, no statistically significant difference was found across the regions in our study. In addition, the relatively low rate in Africa compared to other regions was not expected as the most common secondary headache subtype in the African region following primary headaches was due to infection in a study [40]. These differences may be due to the misdiagnosis of primary headaches due to the limited availability of neuroimaging methods, especially in Africa [23].

### Neuropathies & facial pains

In our study, “Neuropathies & Facial Pains” were observed in 4%. In prior studies, it was observed at 5.2% in India [53] and 3.02% in Türkiye [13], whereas only neuralgia was reported in Iran, at 1.2%; in Burkina Faso 1% [8]. No significant difference was encountered among all the regions in our comparative study.

### Appendix

Especially people with primary headache disorders experience a headache more commonly than the general population in the acute phase of COVID-19 and after vaccination [65]. Knowledge on NOCs regarding the prevalence of headaches related to COVID-19, its complications, and its vaccines are to be enhanced. In a meta-analysis on 104,751 COVID-19 patients, the global prevalence of headache in COVID-19 patients was 25.2% [66]. In our research, headache attributed to COVID-19 was reported with a frequency of 3.5% among patients with headaches. The reason for this difference is that other studies were conducted only on COVID-19-positive patients and were conducted at a time when the effect of the pandemic was more intense. In this study it was found out that headaches attributed to secondary complications of COVID-19 was seen at a rate of 1.2%, and there is still no sufficient evidence to classify headaches attributed to these complications. Furthermore, in our study, headache attributed to COVID-19 vaccines was observed at a rate of 1.5%. In a study executed in the United Arab Emirates, headaches were observed in 9.6% of patients with a higher incidence in female patients after the first dose of the COVID-19 vaccine [67]. In a study conducted on healthcare workers in Türkiye, headache associated with the COVID-19 vaccine was observed at a rate of 30.6% [68]. mRNA COVID-19 vaccines were used for the first time in the context of vaccination, and a meta-analysis study showed that especially mRNA-based SARS-CoV2 vaccines cause more headaches than other SARS-CoV2 vaccines [69]. Vaccine-related headache is not included in the ICHD-3 criteria, and it is important to explore this case within the candidate criteria. Ekizoglu et al. suggested that COVID-19 vaccine-related headache is a different and straightforward secondary headache entity compared to COVID-19-related headaches as well as pre-existing primary headaches of individuals. In addition, some definitions of headache, such as concussion, burning, and stabbing, did not fully comply with those used in the ICHD criteria leading to some difficulties in classification [68].

### Implications

We can list the strengths of our work as follows.

- Synchronized evaluation of patients in different geographic locations by neurologists with a clear protocol
- Delivering preliminary training on ICHD-3 criteria to physicians before the study,
- Including not only outpatients but also patients referred from emergency and other services within the work experience of neurologists are to be evaluated.
- Including regions that represent a significant part of the world’s geography and are not frequently represented in previous studies.
- Revealing the significant difference in the pain severity in different countries by adhering to the same scale.
- Reporting the current frequencies of headaches related to COVID-19 disease, its secondary complications, and its vaccine-related headaches in NCs in different parts of the world.

### Limitations

Although this study is carried out in large geography, more countries and centers are required to represent the relevant regions fully. Although it is a disadvantage that factors such as race, sociocultural level, and education that may affect the analysis were not questioned in the study, it is inferred that the individuals included in the survey generally represent ethnicity in the relevant regions. Since our research is hospital-based, it is not appropriate to generalize its results to the population. Besides, there is easy access to different subspecialties in Iran, especially in big cities, so headache sufferers usually go to headache specialists. Therefore the recorded cases from centers in Iran may not precisely reflect the rate of headache patients in the general clinics there.

## Conclusion

One out of every three patients who applied to neurology clinics complained of headaches. Compared to previous studies, the increase in the rate of patients with primary headaches among patients with headaches is remarkable. Pain intensity was lower in the African region compared to the other regions. Lifetime pain duration is significantly longer in the Middle East than in the other regions as it is in Türkiye than in Africa indicating various motives such as healthcare system regulations and sociocultural, economic, and educational factors.

Migraine headaches are more common in Africa than in Türkiye and Asia and in the Middle East than in Türkiye. TTH has been observed at a higher rate in Türkiye than in Africa and the Middle East. MOH is an increasing problem in all regions and is the most common type of secondary headache. Although this study reveals that the diagnostic capacity of the ICHD-3 criteria is high, it is valuable as it supports the evaluation of some additional criteria in future classifications bearing in mind the prevalence of increased headaches in NCs after the COVID-19 pandemic is important for developing diagnosis and treatment strategies.

## Supplementary Information

**Additional file 1.**

## Data Availability

All data and materials generated in this study are available upon request.

## Abbreviations

ICHD: International Classification of Headache Disorders
NC: Neurology Clinic
COVID-19: Coronavirus Disease 2019
NVS: Numbered Visual Scale
TTH: Tension-Type Headache
TACs: Trigeminal Autonomic Cephalalgias
MOH: Medication-overuse headache
F: M: Female: Male
NOC: Neurology Outpatient Clinic
OC: Outpatient Clinic
NOHC: Neurology Outpatient Headache Clinic
AA: African Americans
