# Supplementary material for: Cross-sectional, hospital-based analysis of headache types using ICHD-3 criteria in the Middle East, Asia, and Africa: the Head-MENAA study
Source: J Headache Pain. 2023 Mar 13;24(1):24. doi: 10.1186/s10194-023-01555-8 (PMC10010217; doi:10.1186/s10194-023-01555-8)
Supplement: Supplementary file 1 — Additional file 1. [file 10194_2023_1555_MOESM1_ESM.pdf]

The Head-MENAA Study Questionnaire

Dear researcher;

The effect of changing socio-cultural, economic, and health conditions, especially after the COVID-19 pandemic, on headaches' type, character, and frequency is a situation that should be investigated and considered.

For this purpose, in this study, which was organized as a multicenter, multinational, we aim to compare headaches' prevalence, types, and characteristics between the Middle East, North Africa, and Asia. This data will be a background for establishing the current situation and creating future telemedicine-based education programs.

Cases will be evaluated on Friday of the first week, Wednesday of the second week, Monday of the third week, Thursday of the fourth week, and Monday of the fifth week in April. All of the diagnoses will be made according to ICHD-3 criteria. To diagnose headache-related COVID-19 or COVID-19 vaccines will be used the World Health Organization (WHO) criteria. Please be careful about a subject that the same patient cannot be recorded repeatedly in the following weeks.

Thank you for your valuable participation in our study.

* Gerekli

1. E-posta *

2. The Center Name *

3. Physician's name *

4. The Region *

   Yalnızca bir şıkkı işaretleyin.

   - Turkey
   - Middle East
   - Asia
   - North Africa

5. The Country (Please, specify in English) *

6. The City *

7. Date *

   Yalnızca bir şıkkı işaretleyin.

   - Friday of the first week (01 Apr 2022)
   - Wednesday of the second week (06 Apr 2022)
   - Monday of the third week (11 Apr 2022)
   - Thursday of the fourth week (21 Apr 2022)
   - Monday of the fifth week (25 Apr 2022)

8. The total number of patients examined *

9. The total number of patients who suffered from headache *

10. Patient number *

11. Initials of the case *

12. Age *

13. Gender *

Yalnızca bir şıkkı işaretleyin.

- Female
- Male

14. Visit room *

Yalnızca bir şıkkı işaretleyin.

- General outpatient
- Neurology outpatient
- Emergency room
- Consultation
- Private office
- Other

15. Duration of headache *

16. Headache frequency of last month *

17. If there is a rare headache disorders, please define in notes section *

18. Mean severity of the headache *

Yalnızca bir şıkkı işaretleyin.

	1	2	3	4	5	6	7	8	9	10	
Mild											Severe

19. The side of the headache

Yalnızca bir şıkkı işaretleyin.

- Unilateral
- Bilateral
- Unilateral but switch sides
- Bilateral dominant on one side

20. The most prominent location of the headache *

Uygun olanların tümünü işaretleyin.

- ☐ Frontal
- ☐ Temporal
- ☐ Occipital
- ☐ Vertex
- ☐ Periorbital
- ☐ Holocranial
- ☐ Other

21. Nausea *

Yalnızca bir şıkkı işaretleyin.

- ◯ No
- ◯ Yes

22. Vomiting *

Yalnızca bir şıkkı işaretleyin.

- ◯ No
- ◯ Yes

23. Photophobia *

Yalnızca bir şıkkı işaretleyin.

- ◯ No
- ◯ Yes

24. Phonophobia *

Yalnızca bir şıkkı işaretleyin.

- ◯ No
- ◯ Yes

25. Accompanying cranial autonomic symptoms *

Uygun olanların tümünü işaretleyin.

- ☐ None
- ☐ Lacrimation
- ☐ Conjunctival injection
- ☐ Eyelid oedema
- ☐ Nasal congestion
- ☐ Rhinorrhoea
- ☐ Ptosis
- ☐ Forehead/facial sweating

26. Please evaluate the effect of the following triggers for your headache attacks? *

Uygun olanların tümünü işaretleyin.

- ☐ None
- ☐ Hunger
- ☐ Dehydration
- ☐ Bright light
- ☐ Weather changes
- ☐ Tiredness/Physical activity
- ☐ Sleep deprivation/oversleeping
- ☐ Emotional stress
- ☐ *Menstruation (only in women)
- ☐ Reading/studying
- ☐ Watching TV or any screen
- ☐ Playing games on PC/cellphone
- ☐ Smells
- ☐ Some specific foods (please write on the text)

27. If some spesific foods trigger your headache, please specify.

28. Aggravation by physical activity *

Yalnızca bir şıkkı işaretleyin.

- ⬭ No
- ⬭ Yes

29. Valsalva effect *

Yalnızca bir şıkkı işaretleyin.

- ⬭ No
- ⬭ Yes

30. Fever *

Yalnızca bir şıkkı işaretleyin.

- ⬭ No
- ⬭ Yes

31. Focal neurological symptom *

Yalnızca bir şıkkı işaretleyin.

- ⬭ No
- ⬭ Yes

32. If there is a focal neurological symptom, please specify *

33. Please choose the headache subtype (If there are more than one headache type; you can click a few of the following. For example; migraine without aura, chronic migraine, and medication-overuse headache)

34. 1. Migraine *

Uygun olanların tümünü işaretleyin.

- ☐ None
- ☐ 1.1 Migraine without aura
- ☐ 1.2 Migraine with aura
- ☐ 1.3 Chronic migraine
- ☐ 1.4 Complications of migraine
- ☐ 1.5 Probable migraine
- ☐ 1.6 Episodic syndromes that may be associated with migraine

35. 2. Tension-type headache (TTH) *

Uygun olanların tümünü işaretleyin.

- ☐ None
- ☐ 2.1 Infrequent episodic tension-type headache
- ☐ 2.2 Frequent episodic tension-type headache
- ☐ 2.3 Chronic tension-type headache
- ☐ 2.4 Probable tension-type headache

36. 3. Trigeminal autonomic cephalalgias (TACs) *

Uygun olanların tümünü işaretleyin.

- [ ] None
- [ ] 3.1 Cluster headache
- [ ] 3.2 Paroxysmal hemicrania
- [ ] 3.3 Short-lasting unilateral neuralgiform headache attacks
- [ ] 3.4 Hemicrania continua
- [ ] 3.5 Probable trigeminal autonomic cephalalgia

37. 4. Other primary headache disorders *

Uygun olanların tümünü işaretleyin.

- [ ] None
- [ ] 4.1 Primary cough headache
- [ ] 4.2 Primary exercise headache
- [ ] 4.3 Primary headache associated with sexual activity
- [ ] 4.4 Primary thunderclap headache
- [ ] 4.5 Cold-stimulus headache
- [ ] 4.6 External-pressure headache
- [ ] 4.7 Primary stabbing headache
- [ ] 4.8 Nummular headache
- [ ] 4.9 Hypnic headache
- [ ] 4.10 New daily persistent headache (NDPH)

38. 5. Headache attributed to trauma or injury to the head and/or neck *

Uygun olanların tümünü işaretleyin.

- [ ] None
- [ ] 5.1 Acute headache attributed to traumatic injury to the head
- [ ] 5.2 Persistent headache attributed to traumatic injury to the head
- [ ] 5.3 Acute headache attributed to whiplash
- [ ] 5.4 Persistent headache attributed to whiplash
- [ ] 5.5 Acute headache attributed to craniotomy
- [ ] 5.6 Persistent headache attributed to craniotomy

39. 6. Headache attributed to cranial or cervical vascular disorder *

Uygun olanların tümünü işaretleyin.

- [ ] None
- [ ] 6.1 Headache attributed to ischaemic stroke or transient ischaemic attack
- [ ] 6.2 Headache attributed to non-traumatic intracranial hemorrhage
- [ ] 6.3 Headache attributed to unruptured vascular malformation
- [ ] 6.4 Headache attributed to arteritis
- [ ] 6.5 Headache attributed to cervical carotid or vertebral artery disorder
- [ ] 6.6 Headache attributed to cerebral venous thrombosis (CVT)
- [ ] 6.7 Headache attributed to other acute intracranial arterial disorder
- [ ] 6.8 Headache attributed to genetic vasculopathy
- [ ] 6.9 Headache attributed to pituitary apoplexy

40. 7. Headache attributed to non-vascular intracranial disorder *

Uygun olanların tümünü işaretleyin.

- [ ] None
- [ ] 7.1 Headache attributed to increased cerebrospinal fluid pressure
- [ ] 7.2 Headache attributed to low cerebrospinal fluid pressure
- [ ] 7.3 Headache attributed to non-infectious inflammatory disease
- [ ] 7.4 Headache attributed to intracranial neoplasia
- [ ] 7.5 Headache attributed to intrathecal injection
- [ ] 7.6 Headache attributed to epileptic seizure
- [ ] 7.7 Headache attributed to Chiari malformation type I (CM1)
- [ ] 7.8 Headache attributed to other non-vascular intracranial disorder

41. 8. Headache attributed to a substance or its withdrawal *

Uygun olanların tümünü işaretleyin.

- [ ] None
- [ ] 8.1 Headache attributed to use of or exposure to a substance
- [ ] 8.2 Medication-overuse headache (MOH)
- [ ] 8.3 Headache attributed to substance withdrawal

42. 9. Headache attributed to infection *

Uygun olanların tümünü işaretleyin.

- [ ] None
- [ ] 9.1 Headache attributed to intracranial infection
- [ ] 9.2 Headache attributed to systemic infection

43. 10. Headache attributed to disorder of homoeostasis *

Uygun olanların tümünü işaretleyin.

- [ ] None
- [ ] 10.1 Headache attributed to hypoxia and/or hypercapnia
- [ ] 10.2 Dialysis headache
- [ ] 10.3 Headache attributed to arterial hypertension
- [ ] 10.4 Headache attributed to hypothyroidism
- [ ] 10.5 Headache attributed to fasting
- [ ] 10.6 Cardiac cephalalgia
- [ ] 10.7 Headache attributed to other disorders of homeostasis

44. 11. Headache or facial pain attributed to disorder of the cranium, neck, eyes, ears, nose, sinuses, teeth, mouth or other facial or cervical structure *

Uygun olanların tümünü işaretleyin.

- [ ] None
- [ ] 11.1 Headache attributed to disorder of cranial bone
- [ ] 11.2 Headache attributed to disorder of the neck
- [ ] 11.3 Headache attributed to disorder of the eyes
- [ ] 11.4 Headache attributed to disorder of the ears
- [ ] 11.5 Headache attributed to disorder of the nose or paranasal sinuses
- [ ] 11.6 Headache attributed to disorder of the teeth or jaw
- [ ] 11.7 Headache attributed to temporomandibular disorder (TMD)
- [ ] 11.8 Head or facial pain attributed to inflammation of the stylohyoid ligament
- [ ] 11.9 Headache or facial pain attributed to other disorder of cranium, neck, eyes, ears, nose, sinuses, teeth, mouth, or other facial or cervical structure

45. 12. Headache attributed to psychiatric disorder *

Uygun olanların tümünü işaretleyin.

- [ ] None
- [ ] 12.1 Headache attributed to somatization disorder
- [ ] 12.2 Headache attributed to psychotic disorder

46. 13. Painful cranial neuropathies and other facial pains *

Uygun olanların tümünü işaretleyin.

- [ ] None
- [ ] 13.1 Trigeminal neuralgia
- [ ] 13.2 Glossopharyngeal neuralgia
- [ ] 13.3 Nervus intermedius (facial nerve) neuralgia
- [ ] 13.4 Occipital neuralgia
- [ ] 13.5 Optic neuritis
- [ ] 13.6 Headache attributed to ischaemic ocular motor nerve palsy
- [ ] 13.7 Tolosa-Hunt syndrome
- [ ] 13.8 Paratrigeminal oculosympathetic (Raeder's) syndrome
- [ ] 13.9 Recurrent painful ophthalmoplegic neuropathy
- [ ] 13.10 Burning mouth syndrome (BMS)
- [ ] 13.11 Persistent idiopathic facial pain (PIFP)
- [ ] 13.12 Central neuropathic pain

47. COVID-19 associated headache *

Yalnızca bir şıkkı işaretleyin.

- ( ) No
- ( ) Yes

48. Headache related to COVID complication *

Yalnızca bir şıkkı işaretleyin.

- ( ) No
- ( ) Yes

49. Headache related to COVID vaccine *

Yalnızca bir şıkkı işaretleyin.

- ( ) No
- ( ) Yes

50. Notes

Bu içerik Google tarafından oluşturulmamış veya onaylanmamıştır.

Google Formlar
